# Deep Learning in Head and Neck Tumor Multiomics Diagnosis and Analysis: Review of the Literature

**DOI:** 10.3389/fgene.2021.624820

**Published:** 2021-02-10

**Authors:** Xi Wang, Bin-bin Li

**Affiliations:** ^1^Department of Oral Pathology, Peking University School and Hospital of Stomatology & National Clinical Research Center for Oral Diseases & National Engineering Laboratory for Digital and Material Technology of Stomatology & Beijing Key Laboratory of Digital Stomatology, Beijing, China; ^2^Research Unit of Precision Pathologic Diagnosis in Tumors of the Oral and Maxillofacial Regions, Chinese Academy of Medical Sciences, Beijing, China

**Keywords:** artificial intelligence, deep learning, head and neck tumors, diagnosis, multi-omics

## Abstract

Head and neck tumors are the sixth most common neoplasms. Multiomics integrates multiple dimensions of clinical, pathologic, radiological, and biological data and has the potential for tumor diagnosis and analysis. Deep learning (DL), a type of artificial intelligence (AI), is applied in medical image analysis. Among the DL techniques, the convolution neural network (CNN) is used for image segmentation, detection, and classification and in computer-aided diagnosis. Here, we reviewed multiomics image analysis of head and neck tumors using CNN and other DL neural networks. We also evaluated its application in early tumor detection, classification, prognosis/metastasis prediction, and the signing out of the reports. Finally, we highlighted the challenges and potential of these techniques.

## Introduction

Head and neck tumors are the sixth most common neoplasms (529,000 new cases annually) and cause 350,000 cancer-related deaths each year (Ferlay et al., 2015; Fidler et al., 2017). Accurate diagnosis and analysis, especially histologic, radiologic, and biological findings, are crucial for therapeutic efficacy and prognosis prediction in precision medicine. A histologic section typically contains 10^6^–10^7^ cells and provides information on cell numbers and the tumor microenvironment (Koelzer et al., 2017). Radiological images contain 50–5,000 quantitative features (Limkin et al., 2017). Therefore, pathologists and radiologists must spend considerable time and effort on the qualitative and quantitative analyses of cell subsets and biomarker expression in a series of images. Also, inter- and intraobserver variations caused by subjective evaluation are inevitable in clinical practice.

Artificial intelligence (AI) was developed in the 1950s (Bini, 2018). The term *big data* was first proposed by the National Aeronautics and Space Administration in 1997 because a dataset is too large to be easily manipulated and managed. Big data refers to extra huge amounts of data integration, storage, analysis, and reuse of various forms of data, such as audio, video, and images. Big data is aimed at generating a large amount of information to assist decision-making and estimate outcomes, at a lower cost in time and labor (Conway et al., 2018). Computer algorithms and well-integrated data are critical for decoding medical big data. Because radiologic images are digitalized, no additional processing is required. Hung et al. (2020) used clinical big data from the SEER database to predict the survival time of patients of oral tumor by machine learning algorithms in 2020. For pathologic diagnosis, the first major step in adopting deep learning (DL) is to use digital whole-slide imaging (WSI) in routine practice (Jeyaraj and Samuel Nadar, 2019). WSI is non-inferior to traditional microscopy for clinical diagnosis (Halicek et al., 2019).

Machine learning (ML), a type of AI, refers to a computer software performing a task by being exposed to the manually crafted features of representative data (Niel and Bastard, 2019). Head and neck tumors are diverse in histology, in the pattern of underlying genetic alterations, and in metabolic signatures, which need a new method to reveal the sophisticated features. An evolution of ML—DL (Helm et al., 2020)—was first applied to the analysis of pathologic images of the head and neck in 2017 (Lu et al., 2017). Several new theories and methods have arisen to facilitate the application of DL in precision medicine, such as backpropagation and multiple layers in the convolution network. The main beauty of DL is to get rid of the handcrafted features and the end-to-end learning procedure. In the same year, DL was applied to radiomics image segmentation of head and neck tumors (Ibragimov and Xing, 2017). As a result of the improvements of computer algorithms and computational pathology, DL now facilitates the identification of benign and malignant tumors, grading of malignant tumors, and prognosis prediction.

Here, we outlined the application of DL algorithms in multiomics to diagnose and analyze head and neck tumors. Because pathological diagnosis of tumors is the gold standard, the application of DL in pathomics is emphasized in the diagnosis section and radiomics in the prognosis section. Finally, we review the challenges and prospects of DL in multiomics diagnosis and analysis.

## Application of DL in Tumor Diagnosis and Multiomics Analysis

**FIGURE 1:**
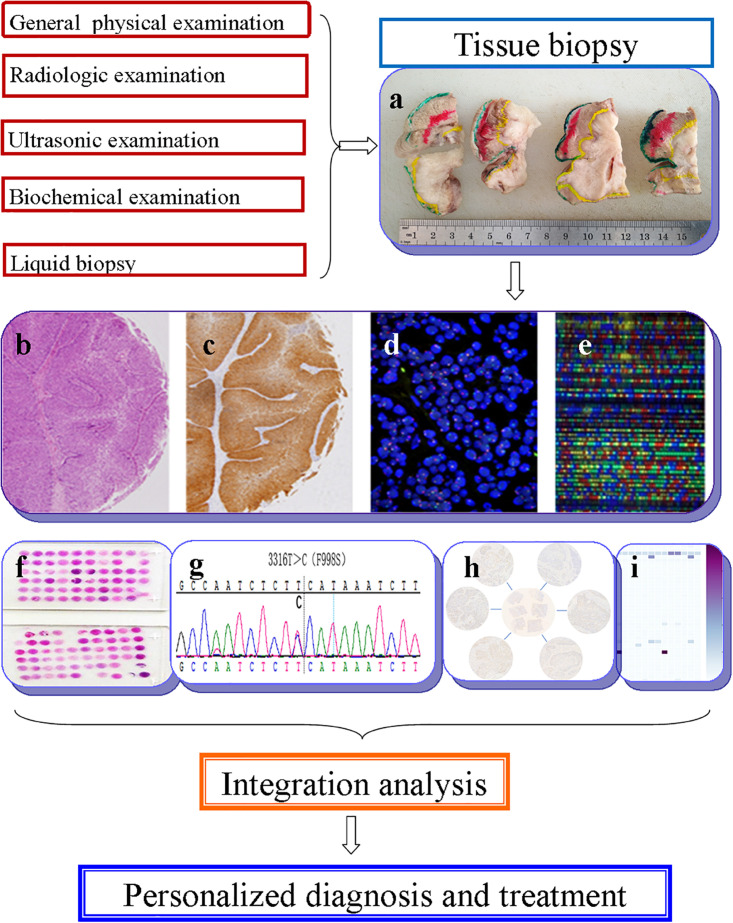
Schematic representation of a synergetic integration of multiomics data. **(a)** Biopsy specimens from head and neck tumor tissue. **(b)** Pathological data. **(c)** Tumor protein data. **(d)** Chromosomal data. **(e)** Gene microarray data. **(f)** Tissue microarray data. **(g)** Gene mutation data. **(h)** mRNA expression data. **(i)** Methylation data.

The term “multiomics” in medicine refers to the combination of multiple sources of information (genomics, transcriptomics, proteomics, metabolomics, radiomics, and pathomics) to provide a deeper understanding of the tumor pathogenesis and lesion nature (Mars et al., 2020; Ye Y. et al., 2020; Figure 1). A schematic representation of a synergetic integration of multiomics data is shown in Figure 1. DL techniques have already been applied for multiomics analysis in various tumors. The identification of tumor origin and essential gene is critical for molecular targeted therapies and accurate treatment and lays the foundation to reveal changes in oncogenic mutation by liquid biopsy. Actually, multiomics is heterogeneous data which is difficult to be comprehensively analyzed. However, the DL network takes this challenge into an opportunity. DL-based multiomics analysis has allowed to classify groups of patients based on a more individual scale in the era of precision medicine. A timeline demonstrating the researches of DL in tumor diagnosis and multiomics analysis is shown in Supplementary Figure 1. Identifying robust survival subgroups of head and neck squamous cell carcinoma (HNSCC) will significantly improve patient outcome. Zhao et al. (2020) established a DL-based disease progression model on 86 HNSCC patients’ data using methylation data, RNA sequencing (RNA-Seq), and miRNA sequencing (miRNA-Seq) from The Cancer Genome Atlas (TCGA). The results of the autoencoder DL model demonstrated that patients were classified into two subgroups with a significant difference in progression-free survival (PFS). The predictability of this model was validated using three independent cohorts. The different biological origin of the tumor tissue has distinct clinical behavior. In practical clinical situations, it is difficult to distinguish between poorly differentiated carcinoma and metastatic carcinomas. Jiao et al. (2020) constructed a multiclass deep learning/neural network (DNN) model to integrate the whole genome sequence and pathomics data to shed light on a comprehensive view of the histological origin of the tumor cells. They evaluated three features, namely mutation distribution, mutation type, and driver gene/pathway. The classifier achieved predictive accuracies of 91% in 24 types of tumors.

Artificial neural network models have been used to investigate the relationship between the symptoms of oral cancer and its prognosis (Tseng et al., 2015). Phillips et al. (2019) used DL models to detect pigmented dermoscopic images, thus improving the accuracy of early melanoma diagnosis. Clinically, it is difficult to differentiate ameloblastomas from keratocystic odontogenic tumors depending only on X-ray. CNN can assist in the diagnosis of ameloblastoma and keratocystic odontogenic tumors based on transfer learning. The sensitivity, specificity, and accuracy were 81.8, 83.3, and 83.0%, respectively. Interestingly, the model performed consistently well, just like skilled experts (Poedjiastoeti and Suebnukarn, 2018). In addition to X-ray research, Fu et al. (2020) used clinical photographic images to predict the early occurrence of oral tumor through a cascaded CNN model. After training by 1,469 samples, the sensitivity and specificity reached 94.9 and 88.7%, separately. This study also provided a non-invasive and highly efficient perspective on oral tumor detection. It was also possible to start providing early treatment immediately. In IDH1 wild-type glioblastomas, methylation modification had a great influence on chemotherapy response and prognosis. Le et al. (2020) used a radiomics-based eXtreme Gradient Boosting (XGBoost) model to predict the IDH1 wild-type patients with O6-methylguanine-DNA methyltransferase (MGMT) promoter methylation status. Nine robust radiomics features were selected based on the *F* score to improve the diagnosis of MGMT methylation status in IDH1 wild-type glioblastomas and predict patient prognosis. Sulfation of the protein S site is an important posttranscriptional modification, which plays a vital role in signal transduction, transcriptional regulation, and cell apoptosis. However, traditional experiments for its biological functions were not timely due to its rapid degradation. Do et al. (2020) used a DL network to predict protein phosphorylation S site based on the proteomics data. The DL network was also used to predict the function of fertility-related proteins in infertility patients and paved the way for a better understanding of the function of fertility proteins (Le, 2019). Therefore, the integration of DL, image analysis, and big data enables the evaluation of tumor biological behavior and, hence, facilitates diagnosis, personalized treatment, and survival prediction.

## Head and Neck Tumor Multiomics Analysis

### Multiomics Analysis in Early Detection of Tumors

The global incidence of head and neck cancer is 1.3 million annually. The risk factors for head and neck tumors are chewing tobacco, local irritation, smoking, alcohol abuse, human papillomavirus infection, etc. It is necessary to monitor the occurrence of oral cancer in high-risk groups. Early diagnosis could reduce the mortality rate to 70% at present (Erickson et al., 2018). Also, DL could enable regular follow-up of high-risk groups. Moreover, DL methods can be applied not only to low-level tasks (e.g., recognition, detection, and segmentation) but also to more advanced tasks (e.g., selection of the optimal treatment and prediction of prognosis).

As we know, routine tissue biopsies are invasive. Although it is safe, some risks may be brought in rare cases and non-invasive biopsy comes into being. In non-invasive modalities, a large number of images appeared combined with training of DL networks based on oral clinical examinations and histological findings, which would assist in the evaluation of precancerous and cancerous lesions. The human eyes and cameras capture three color channels—red, green, and blue. Hyperspectral imaging involves multiple wavelengths, enabling the identification of cancerous and normal tissue by optical biopsy. Halicek et al. (2017) trained a CNN to identify hyperspectral images of squamous cell carcinoma (SCC). The reported accuracy, sensitivity, and specificity of the training set were 81, 81, and 80%, respectively. The hypercube contained 91 spectral bands, ranging from 450 to 900 nm with a 5-nm spectral sampling interval. Similarly, confocal laser endomicroscopy (CLE) allows real-time visualization of epithelium *in vivo* and enables early diagnosis of oral cancer and prediction of the prognosis. In 2007, Soo et al. reported the application of CLE for the diagnosis of oral SCC (OSCC) (Thong et al., 2007). Subsequently, Nathan et al. applied CLE to detect head and neck precancerous lesions; the sensitivity and specificity for the diagnosis of oral epithelial dysplasia were 85.7 and 80.0%, respectively (Moore et al., 2016). Aubreville et al. (2017) proposed an automatic framework for the application of CLE to detect cancerous lesions by CNN. In the proteomics research of head and neck tumors, Ni et al. (2015) used artificial neural networks to screen out proteins which were related to lymph node metastasis using the proteins extracted from the saliva of OSCC patients.

Radiomics is also used as one of the non-invasive clinical examinations. Ren et al. (2018) used the least absolute shrinkage and selection operator (LASSO) logistic regression to extract features from magnetic resonance images (MRI) of head and neck SCC to predict the histological grade before surgery. Subsequently, the same method was applied in floor-of-the-mouth and tongue SCC by Ren et al. (2020). Computed tomography (CT) can also be used to predict the histological classification before surgery by kernel principal component analysis (KPCA) and the random forest classifier (Wu et al., 2019). Mukherjee et al. (2020) performed principal component analysis and regularized regression to predict tumor grade, extracapsular spread, perineural invasion, lymphovascular invasion, and human papillomavirus infection status. The accuracy, sensitivity, and specificity of the model were 0.72, 0.83, and 0.48, respectively. DL is also applied in radiomics. Ye J. et al. (2020) used a CNN model for histological classification of head and neck tumors; the accuracy, sensitivity, and specificity were 0.79, 0.71, and 0.85, respectively. The utility of AI for the analysis of head and neck pathologic sections and radiologic images is summarized in Tables 1, 2.

**TABLE 1:** Summary of deep learning models for H&N tumor Pathomics analysis.

Topic	H&N tumor subtype	Task	Model	References
H&N tumor detection and classification	H&N squamous cell carcinoma (HNSCC)& thyroid carcinoma	Malignant vs. non-malignant classification	CNN	Witjes et al., 2018
	OSCC	According to the keratin pearl to classify the high-grade or low-grade OSCC	CNN	Das et al., 2018
	OSCC	Malignant vs. non-malignant classification in CLE image	CNN	Aubreville et al., 2017
	Oral tumor	Malignant vs. benign vs. precancerous classification	CNN	Jeyaraj and Samuel Nadar, 2019
H&N tumor segmentation	OSCC	Tumor margin detection and segmentation	CNN	Halicek et al., 2018
	OSCC	Segmentation the boundary of tumor and normal tissue	CNN	Halicek et al., 2017
	TSCC	Tumor margin detection and segmentation	CNN	Yu et al., 2019
	OSCC	Quantity nuclear morphology to stratify patients of high or low risk	CNN	Lu et al., 2017
	OSCC	Based on clinic-hiotopathology features to predict patient’s outcome	DL	Kim et al., 2019
	OSCC	Quantity tumor infiltrating lymphocytes to predict the patients’ outcome and treatment response	CNN	Shaban et al., 2019

*H&N, head and neck; OSCC, oral squamous cell carcinoma; CNN, convolution neural network; CLE, confocal laser endomicroscopy; DL, deep learning.*

**TABLE 2:** Summary of machine learning and deep learning models for H&N tumor Radiomics analysis.

Topic	H&N tumor subtype	Task	Model	References
H&N tumor prognosis	H&N squamous cell carcinoma (HNSCC)	Loco-regional control (LRC)	PCA	Bogowicz et al., 2019b
	head and neck cancer (HNC)		Z-Rad radiomics software	Bogowicz et al., 2019a
	Locally advanced head and neck cancer		Free LifeX software package	Cozzi et al., 2019
	HNSCC	Overall survival (OS)	RadiomiX Discovery Toolbox.	Keek et al., 2020
			In-house built Accurate tool	Martens et al., 2020
			LASSO	Yuan et al., 2019
			PCA	Mes et al., 2020
	H&N tumor		Random survival forests (RSF) and random forest (RF)	Leger et al., 2019
			Velocity AI v3.0.1 software and Imaging Biomarker Explorer and k-medians	Tosado et al., 2020
			Matlab R2018b	Lv et al., 2020
			Z-Rad software and Hierarchical Clustering	Bogowicz et al., 2020
			IBEX, an open-source radiomics tool	Ger et al., 2019
	Aryngeal squamous cell carcinoma		LASSO	Chen L. et al., 2020
Biologic markers prediction	Oropharyngeal squamous cell carcinoma	HPV status prediction	In-house developed software, using Matlab 2014a	Leijenaar et al., 2018
	Oropharyngeal cancers	HPV status prediction	PCA	Bagher-Ebadian et al., 2020
	HNSCC	HPV status and T-cell infiltration prediction	Unsupervised consensus clustering and PCA	Katsoulakis et al., 2020
H&N tumor recurrence and metastasis	HPV-related Oropharyngeal Carcinoma	Distant metastasis	Unpublished MATLAB code	Kwan et al., 2018
	H&N tumor	Metastatic lymph nodes	Naive Bayes, and k-nearest neighbor classifiers	Tran et al., 2019
	Locally advanced head and neck cancer	Recurrence	Random forest	Beaumont et al., 2019
	H&N tumor	Lymph node metastasis	3-dimensional CNN	Zhou et al., 2018; Chen et al., 2019
	Papillary thyroid carcinoma		SVM	Liu et al., 2019
	H&N tumor		Matlab	Zhai et al., 2020

*H&N, head and neck; PCA, principal component analysis; LASSO, the least absolute shrinkage and selection operator; SVM, support vector machine.*

### Multiomics Analysis in Tumor Detection, Segmentation, and Classification

**FIGURE 2:**
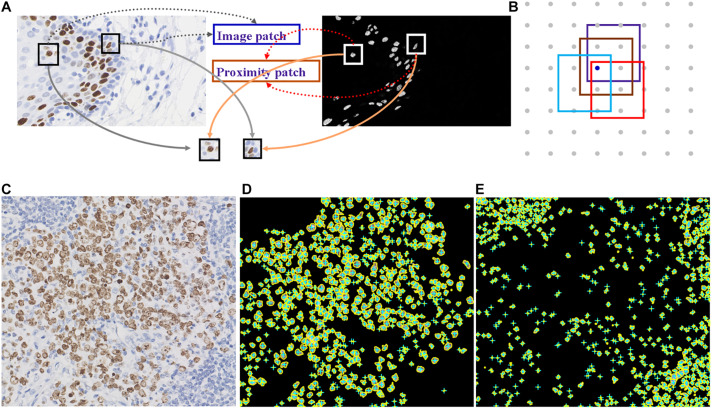
Example of segmentation of nuclei in a cell layer on WSI images. **(A,B)** Illustrations of nucleus segmentation based on the DL model. **(C)** The original image of immunohistochemical staining. **(D)** Nucleus segmentation for immunohistochemistry-positive cells. **(E)** Nucleus segmentation for immunohistochemistry-negative cells.

**FIGURE 3:**
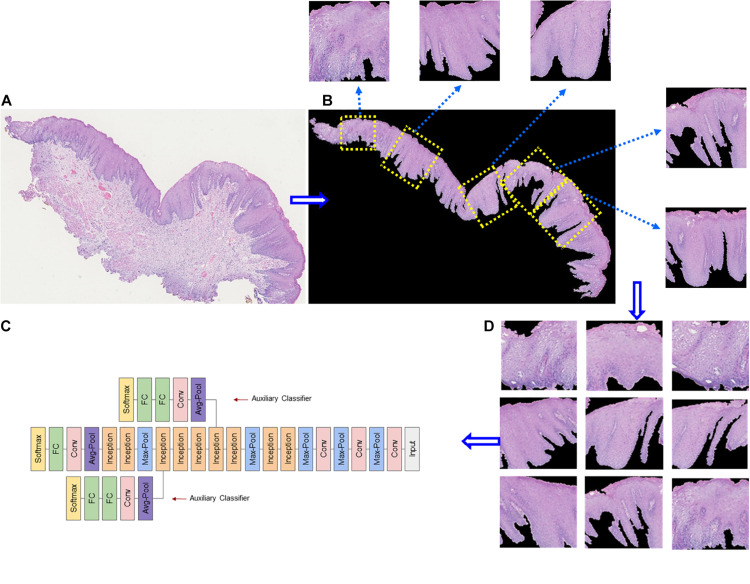
Example of epithelial segmentations on WSI images. **(A)** The original image. **(B)** Black curves indicate the segmented boundary of the epithelium. **(C)** Input patches for training the DL network. **(D)** Schematic of a deep learning framework.

Deep learning is suitable for digital pathology (DP)-related image analysis tasks, such as detection (e.g., lymphocyte), segmentation (e.g., nuclei and epithelium), and classification (e.g., the tumor subclass). Figures 2, 3 demonstrate an example of epithelial segmentations on WSI images and an example of segmentation of nuclei in a cell layer on WSI images. Different from ML, which classifies handcrafted features (Das et al., 2018), DL takes an agnostic approach by combining feature extraction and the interest region analysis.

**FIGURE 4:**
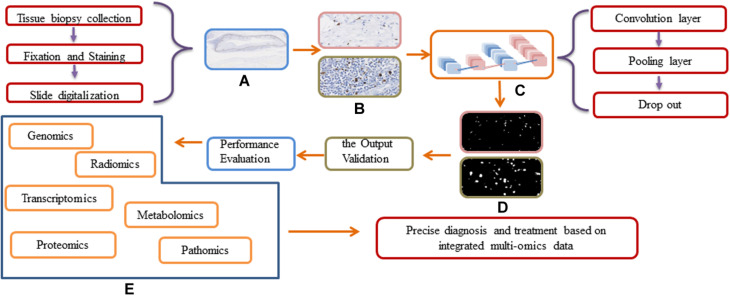
Workflow and general framework for DL approaches in digital pathology. **(A)** The original WSI image. **(B)** Different resolution of WSI images before input the DL network. **(C)** The Schematic of a deep learning framework. **(D)** The output of different resolution of WSI images. **(E)** The integration of multi-omics data.

In head and neck tumor diagnosis, the morphology of heterogeneous cell types needs to be evaluated. This can be formulated as a pixel-wise detection task. The detection tasks frequently align with the classification tasks, and the algorithms learn the weighted parameters of the feature map. The algorithms map clusters of similar features to the output labels. The workflow for DL approaches in digital pathology is shown in Figure 4. In traditional ML, the workflow is comprised of two steps: detection and classification. For instance, Lewis et al. (2014) developed an approach to quantify automatically the morphologic features used for the classification of aggressive or indolent p16-positive oropharyngeal SCC. A cluster cell graph was generated to evaluate the spatial distribution of mitotic cells, and a random forest (RF) decision tree and SVM were used to classify features. The accuracy of the model was 87.5% (140 patients). However, it may not be applicable to other situations because of the small training dataset and the overfitting problem. Moreover, the accuracy of DL is unsatisfactory. Several proposed DL models for detecting head and neck tumors overcome the abovementioned shortcomings. Aubreville et al. (2017) trained a DL model to detect an image patch from doubtful OSCC cases. Overall image recognition had an area under the curve (AUC) of 0.96 and a mean accuracy of 88.3% (sensitivity 86.6% and specificity 90%).

Halicek et al. trained a deep CNN to identify surgical margins accurately in hyperspectral images. Additionally, an end-to-end DL network can simultaneously detect and enumerate mitotic cells (Jimenez and Racoceanu, 2019). In the above reports, DL was only used for dimension reduction or feature extraction. It also may be a classifier to perform classification (Boldrini et al., 2019). Usually, the end-to-end DL approach performed better than none end-to-end learning. However, as for pathomics, one end-to-end DL model cannot perform multiple tasks simultaneously. Lei et al. (2019) trained a convolutional neural network by DL to extract mitosis features automatically and proposed a network to determine the location of all mitotic cells. This approach showed an unexpectedly high accuracy in the International Conference on Pattern Recognition (ICPR2012) mitosis detection test dataset. The remaining challenges include accurate identification and enumeration of mitotic cells in two-dimensional (2D) digital histology images. The imaging of three-dimensional (3D) tissues in 2D results in loss of spatial information. Radiomics enables enhanced 3D assessment of tumor growth by quantifying changes in tumor cellularity and angiogenesis. Radiomics analysis also shows potential for the accurate quantification of heterogeneity and outcome prediction.

Microscopic cell structure recognition is emphasized in pathomics. A common strategy for detecting cells or nuclei is to train a CNN classifier as a pixel classifier, in which a patch centered on the object of interest is used to train the network under supervised conditions. Trained CNN models typically comprise two classifiers (yes or no) and can be applied to WSI in a sliding window to detect all histological components of interest and output a probability map, where each pixel is transferred to a probable value. Therefore, in principle, the target objects can be located by finding a local maximum in the generated probability map. Fully convolutional networks can share calculations on sliding windows. After completing nuclear or mitotic detection tasks, it begins counting or extracting quantitative indicators in WSI. The algorithm is built on mapping an input image patch to a density map, which is used to estimate the number of cells in the original image.

Deep learning also plays an important role in the analysis of tumor microenvironment characteristics (TMC). The crucial step in TMC analysis is segmenting different types of tissue and cell structures in pathology images. Tumor cells can be classified into parenchymal and stromal cells. Niranjan and Sarathy (2018) reported the ratio of tumor to stroma (TSR) as a reliable histologic predictor of overall survival and outcome in OSCC. In a cohort of 60 OSCC patients, the 3-year overall survival (OS) and disease-free survival (DFS) rates of patients with >50% intratumor stroma had been shown to be better than the patients with <50% intratumor stroma.

The segmentation task is more difficult than mitosis detection because parenchyma segmentation can be labeled by experts at lower magnifications. However, stroma (e.g., lymphocytes, macrophages, fibroblasts, etc.) must be analyzed at high magnification. Indeed, × 40 magnification performed better than × 20 magnification for nucleus segmentation. By contrast, epithelium segmentation is typically more precise by experts at × 20 than at 40 × magnification, as indicated by a higher accuracy and *F* score (Janowczyk and Madabhushi, 2016). To remedy this drawback, the fully convolutional network (FCN) and UNet were designed to accept discretional size as an input and product proportionate-sized outputs by removing all fully connected layers and introducing unsampled layers to offset the shortcomings of downsampling in CNN (Zhou et al., 2019). Considering that head and neck tumors are heterogeneous and complex, segmentation may involve varied anonymous anaplastic cells and then can be achieved by data augmentation. Halicek et al. (2018) trained a CNN to segment the tumor and normal tissue of OSCC with 81% accuracy, 84% sensitivity, and 77% specificity. The sensitivity and specificity of FCN for cervical tumor segmentation on 3D FDG-PET images were 88 and 98%, which were markedly superior to CNN. Unfortunately, FCN has not been used for segmentation of pathologic images of head and neck tumors. Moreover, tumor segmentation accuracy is associated with loss function. Now, the well-known loss function is cross-entropy loss. A new loss function, class-wise DSC loss, for training the segmentation network of colonoscopy pathology images was presented by Feng et al. (2020).

### Multiomics Analysis in Tumor Prognosis and Metastasis

The high heterogeneity and complexity of head and neck tumor pathology images hamper the prediction of outcomes only by TNM stage. In recent years, more and more scholars have been interested in the potential of DL networks for predicting postoperative outcomes. The applications of radiomics to predict overall survival, biomarker status, recurrence, distant or local metastasis, and lymph node metastasis are summarized in Table 2. Tixier et al. used the Genomica software to analyze PET and transcriptomics data of 45 patients with locally advanced head and neck cancer. They applied a fuzzy locally adaptive Bayesian (FLAB) algorithm to assess the associations between radiomics features (a total of 28 image biomarker standardization initiative-compliant radiomic features) (Zwanenburg et al., 2020) and alterations of biological pathways (e.g., extracellular matrix organization, cell cycle, signal transduction, cell cycle, etc.). The results demonstrated that FDG-PET radiomic features were associated with cell cycle, DNA repair, extracellular matrix organization, immune system, metabolism, and signal transduction pathways, providing a thorough understanding of genetic mutations and minimizing the costs (Tixier et al., 2020). Zhu et al. (2019) integrated the genome-wide multiomics data of 126 patients with head and neck SCC with CT imaging data and found the significant association between genomic characteristics and CT features. The use of DL together with sophisticated biomarkers can significantly improve prognostic and predictive accuracy. Subsequently, the DL-extracted imaging features of morphology structure on digitized H&E-stained tissue sections have been used for risk stratification of head and neck tumor patients. Patients with p16-positive human papillomavirus-related oropharyngeal SCC have a more favorable prognosis than those negative for P16 (Ali et al., 2013). Lewis et al. (2014) used a typical ML approach (the random forest decision tree) to extract nuclear morphologic features and predict progression. Before the advent of DL, improvement of prognosis was evaluated by multifactor analysis, conventional logistic regression, and Cox analysis in traditional ML models. However, the absence of a decision rule and linear combinations of covariates hampered the prediction of outcomes. DL-based survival prediction has improved predictive accuracy and, together with nonlinear algorithms, will facilitate precision medicine. Therefore, it is suitable for predicting the survival of inpatients (Tan et al., 2016). Tseng et al. constructed a DNN to predict the survival of patients with oral tumors using clinical variables and histopathological features. It was suggested that the DNN model established by data mining was superior to logistic regression in terms of both training accuracy and cross-validation accuracy. Brennan et al. (2017) used an unsupervised cluster analysis method to interpret the genomics and epigenetics data of morphologically atypical head and neck SCC and found CpG island methyl groups in atypical SCC. Therefore, novel prognostic factors, such as genetic mutations and molecular markers, combined with clinicopathologic and radiologic features and a multi-nonlinear DL network would yield optimal results.

Proteomics and transcriptome have also been used to study lymph node and distant metastasis and recurrence of SCC patients. Onken et al. (2014) used an unsupervised clustering algorithm to extract transcriptome signature predicting distant metastasis in oral tumor over four SCC datasets. Xu et al. (2014) applied a ML approach called maximum relevance minimum redundancy algorithm to a set of transcriptome data generated from papillary carcinoma and anaplastic carcinoma for differential diagnoses. The lung is the most common site of distant metastasis of OSCC. Primary SCC can also occur in the lung. Through supervised learning and analysis of proteomic data, Bohnenberger et al. (2018) found the vital difference of protein characteristics between lung metastatic head and neck SCC and primary lung SCC. Their data provided reference information for the origin of lung SCC. Carnielli et al. (2018) used histological morphology-oriented proteomics analysis of the protein expression in tumor islands and stroma to forecast the possibility of tumor recurrence and lymph node metastasis. Six ML approaches were used by Kaddi and Wang (2017) to analyze proteomics and transcriptome data, including KNN, SVM, naive Bayes, DT, AdaBoost, and RF. It was shown that the prognostic model based on both transcriptome and proteomics data had better predictive performance than transcriptomics or proteomics alone.

### Diagnostic Reports: Automatic Extraction of Tumor Information

Zhang et al. (2017) developed MDNet, which generated pathological reports by directly mapping pathology images with simultaneous retrieval of pathology images according to symptom descriptions. MDNet added a language network to the original image model. Integration of a language model with the multiscale features proposed by the image model allowed the identification of critical image features and enabled the direct mapping from words to pixels. Changes in the size or density of nuclei and epithelial thickness may indicate neoplastic invasion. However, these discriminant imaging features were not directly supported to generate a diagnostic report. MDNet allowed direct multimodal mapping from medical images and diagnostic reports. Mimicking diagnosis by pathologists, long short-term memory (LSTM) networks were used to generate semantic information as a language model. The LSTM was a representative gated RNN that controlled the forget gate and input gate to emphasize or forget some weights. It could reduce the problem of multiple layers and vanished gradient multilayers from input to output.

### Radiogenomics Analysis for Radiotherapy Patients

Radiogenomics is a computational nomenclature which identifies correlations between radiomics imaging features and genomics or proteomics data. These imaging feature correlations can be used to predict a tumor’s molecular profile in clinical radiomics data (West and Rosenstein, 2010). Radiogenomics has two goals: i) discover the patients who are more likely to develop radiotherapy complications based on molecular data and (ii) analyze the targeted molecular pathway responsible for radiotoxicity in radiation-induced normal tissue (Kerns et al., 2014). Postoperative radiotherapy is an effective treatment for head and neck tumors. The existence of radiosensitivity and radioresistance may be related to genetic factors partially. The remaining differences between individuals were caused by differences in treatment (radiation dose), physical habits, and random factors (Rattay and Talbot, 2014). Werbrouck et al. (2009) reported that the DNA repair genes *XRCC3* and *Ku70* were connected to the intensity of dysphagia after radiotherapy in H&N tumor in 2009. For the study of postradiotherapy mucositis, Yang et al. (2020) sequenced and located the gene expression in 1,497 patients with postoperative radiotherapy. They found that 64 target genes were enriched in the process of telomerase regulation, which confirmed the importance of telomere function in the development of radiation-induced adverse reactions. The combination of PET-based spatial radiation features and sequencing data provided a new perspective for further revealing the spatial heterogeneity of tumors (Clasen et al., 2020). Furthermore, the predictive analysis of gene expression and cellular and molecular expression can be provided from a non-invasive point of view, based on the radiological characteristics and gene differential expression data of head and neck tumors obtained from the TCGA and TCIA databases (Katsoulakis et al., 2020).

## Difficulties and Expectation

AI is highly dependent on a robust and large database, but the database of pathological slides of head and neck tumors has not been established yet. Apart from the hardware needed to set up the database, setting up an autoprocessed image database is also needed. When there were images captured from clinical cases, the database could have the images with their properties at the same time, which would help in further analysis. As time goes on, the database could grow by itself (Ibrahim et al., 2020). The low-quality images are also a problem for DL analysis. According to a jointed framework proposed by Chen J. et al. (2020), a novel transfer learning strategy called channel fusion transfer learning and a deep super-resolution framework called SRFBN+ were dedicated to generating higher-resolution slice images with lower-resolution ones as input. The most successful application of DL in medical image analysis has been in supervised learning. On the other hand, the rarity of pathologists added the extra difficulties in data cleaning and labeling, while the high heterogeneity of head and neck tumors means that many rare tumors need to be accurately labeled.

A crucial step is to avoid subjective and sample biases in the training sets as the quality of the output depends on the quality of the input data (Oakden-Rayner, 2020). So, establishing a unified standard to normalize the image input in the network by multi-institution datasets can not only reduce the bias from the samples and the bias caused by inconsistent diagnostic from the physicians but also fully fit and train the model to reduce overfitting and reduce to a maximum the highly opaque nature of medical image (Martorell-Marugán et al., 2019). However, current DL algorithms are mainly trained on a small dataset from a single center (Jiang et al., 2020). The limited availability of well-characterized and adequately stored clinical tumor and non-tumor samples is a major challenge in proteomics and genomics researches (Matta et al., 2010).

For the algorithms themselves, the tendency has been to propose new algorithms rather than optimize those already used, leading to the conclusion that there is no improvement of some subdomain algorithms. In addition, due to the limitations of, for instance, data and computational power, the improvement of algorithms must take into account various trade-offs. Additionally, some studies used a non-open-source code or a non-open-source model, such as an in-house developed model, hampering model verification in other types of tumors (Parmar et al., 2015; Leijenaar et al., 2018). A flowchart demonstrating the relationship for the subsection of difficulties and expectation of DL in tumor diagnosis and multiomics analysis is shown in Supplementary Figure 2.

### Difficulties Related to Unified Evaluation Standards

The lack of unified innovation evaluation standards in AI has led to some exaggeration of the improvements achieved. This can be overcome by a variety of methods, e.g., an open-source or source model. Unifying evaluation standards is difficult but is possible for some mature domains. The relevant data management domains are as follows: (i) administrative standards, (ii) patient privacy protection standards, and (iii) intellectual property protection standards. The establishment of data management standards would allow access to diverse anonymized imaging datasets. Technical standardization cannot resolve all of the issues described in this review. The use of different image normalization or style conversion methods (e.g., rotating, cropping, zooming, and image histogram-based modifications) for preprocessing could overcome the technical obstacles.

### Difficulties in Image Analysis

The architectures of CNNs have been especially powerful for computer vision, particularly in image interpretation and procession. WSI combined with DL algorithms for tumor detection, classification, and prognosis prediction has played an ever-increasing part in supporting pathologists in clinical assessments. The main components of CNN are convolutional layers and pooling layers. Although CNN has advantages in the processing of object detection, it has notable drawbacks: (1) both the training and the detection process is considerably time-consuming and (2) the normalization method would lead to lose some discriminative details. FCN is suitable for image segmentation at the pixel level. It consists of convolution and deconvolution layers, which can accept input images of any size and retain the spatial information of the original input lines. The major disadvantages of FCN may be that it is noisy and contains redundant information, requiring a huge number of reliable samples. To overcome the issues mentioned above, more novel architectures (e.g., UNet++, SegNet, and ENet) based on FCN or CNN have been proposed for image segmentation. Pan et al. (2020) proposed a DL model based on the architectures of FCN to automatically recognize lymph node metastasis of esophageal SCC. Compared with previous studies focused on the isolated tasks in the analysis of pathology and radiology images, the integration of independent DL models into a general model would be beneficial (Wang et al., 2019). It was also anticipated that biological pathways and gene regulation networks would be incorporated into prediction models, improving their performance and interpretability. For multimodal learning, collecting data from the required modalities simultaneously could be problematic. A slight disturbance to the inputs of multimodal can influence the stability of CNN. Lin et al. (2020) trained a multiscale activity transition network to provide an activity state pyramid consisting of multiscale recurrent neural networks to capture the accurate feature of input. Transfer learning is frequently used and is an effective pretraining strategy. The fusion of different modal representations is the key point of a multimodal task. Specific fusion operations are based on an attention mechanism or bilinear pooling. In practice, fusion operations are often diverse and complicated (Mormont et al., 2020).

### Integration of Multiomics Data and Precision Medicine

Now, DL algorithms still have several difficulties of integrating multiomics data or various sources of information such as pathology images and electronic medical records. The use of DL to accomplish simple tasks can yield useful results. Furthermore, complex datasets, abundant neural network architecture, and adequate DL methods are anticipated to provide useful information for precision medicine. Pathomics and radiomics are crucial components of multiomics, which also include genomics, transcriptomics, proteomics, and metabolomics information. Although there are still some limitations that restricted the direct clinical usage of multiomics analysis, there is still an increasing effort in solving the drawbacks to provide promising applications. The increasing number of omics datasets is fuelling the quantitative analysis of biological specimens at the gene, cell, and tissue levels. It will generate novel hypotheses on the molecular mechanisms of tumor development and progression for guiding precise diagnosis and treatment.

## Author Contributions

BL: conceptualization, writing—review and editing, supervision, and funding acquisition. XW: formal analysis, data curation, and writing—original draft. Both authors contributed to the article and approved the submitted version.

## Conflict of Interest

The authors declare that the research was conducted in the absence of any commercial or financial relationships that could be construed as a potential conflict of interest.

## Supplementary Material

Supplementary Figure 1 A timeline demonstrating the DL researches in tumor diagnosis and multi-omics analysis.

Supplementary Figure 2 A flow chart demonstrating the difficulties and expectation of DL in tumor diagnosis and multi-omics analysis.
