# Supplementary figures and images for: Deep Learning in Head and Neck Tumor Multiomics Diagnosis and Analysis: Review of the Literature
Source: Front Genet. 2021 Feb 10;12:624820. doi: 10.3389/fgene.2021.624820 (PMC7902873; doi:10.3389/fgene.2021.624820)

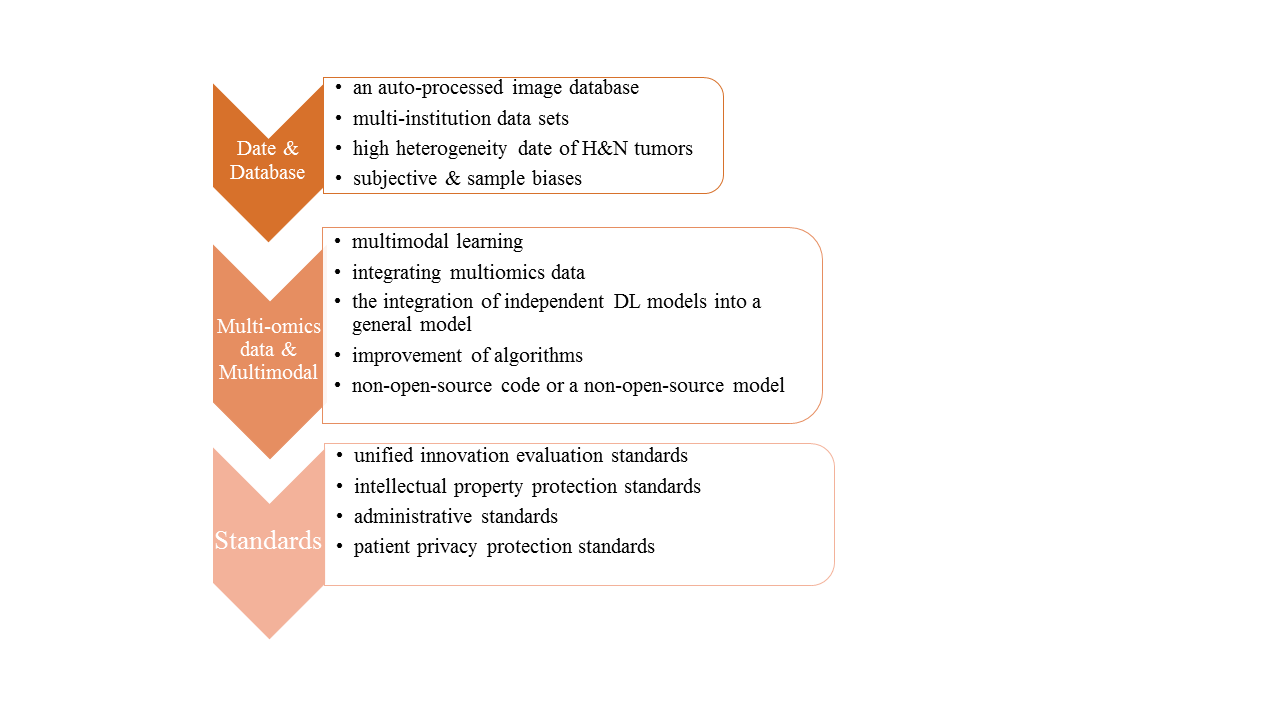

Supplement: Supplementary Figure 1 — A timeline demonstrating the DL researches in tumor diagnosis and multi-omics analysis. [file Image_1.TIF]

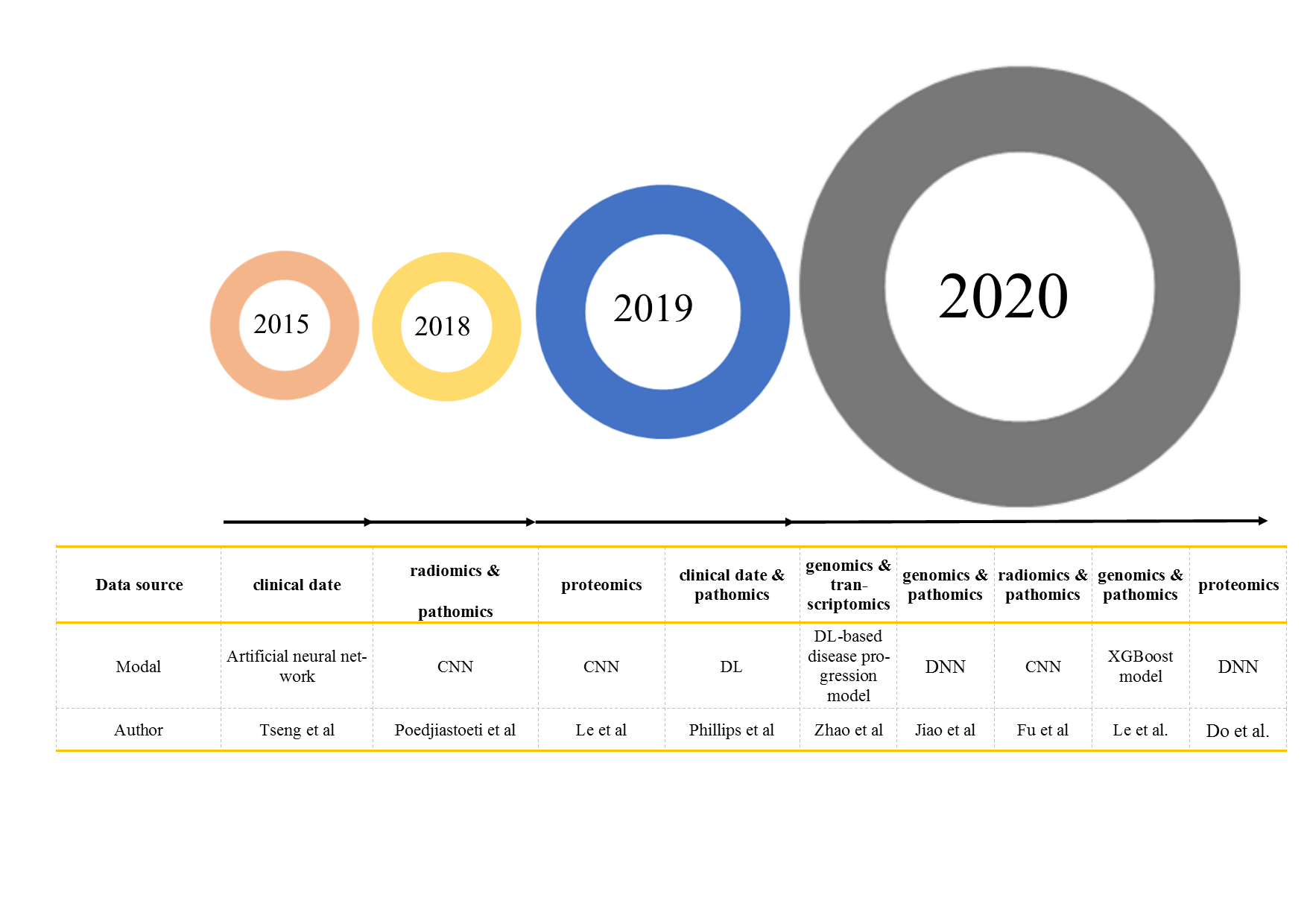

Supplement: Supplementary Figure 2 — A flow chart demonstrating the difficulties and expectation of DL in tumor diagnosis and multi-omics analysis. [file Image_2.TIF]
